# Accuracy of Xpert® MTB/RIF Ultra test for posterior oropharyngeal saliva for the diagnosis of paucibacillary pulmonary tuberculosis: a prospective multicenter study

**DOI:** 10.1080/22221751.2022.2148564

**Published:** 2022-12-12

**Authors:** Peijun Tang, Rongmei Liu, Lin Qin, Ping Xu, Yu Xiong, Yunfeng Deng, Zizheng Lv, Yuanyuan Shang, Xinghui Gao, Lin Yao, Ruoyu Zhang, Yanjun Feng, Caihong Ding, Hui Jing, Liang Li, Yi-Wei Tang, Yu Pang

**Affiliations:** a Department of Tuberculosis, The Fifth People's Hospital of Suzhou, The Affiliated Infectious Diseases Hospital, Suzhou Medical College of Soochow University, Suzhou, People’s Republic of China; b Department of Tuberculosis, Beijing Chest Hospital, Capital Medical University/Beijing Tuberculosis & Thoracic Tumor Research Institute, Beijing, People’s Republic of China; c Department of Bacteriology and Immunology, Beijing Chest Hospital, Capital Medical University/Beijing Tuberculosis & Thoracic Tumor Research Institute, Beijing, People’s Republic of China; d Department of Endoscopic Diagnosis & Treatment, Beijing Chest Hospital, Capital Medical University/Beijing Tuberculosis & Thoracic Tumor Research Institute, Beijing, People’s Republic of China; e Department of Clinical Laboratory, The Fifth People’s Hospital of Suzhou, Infectious Disease Hospital Affiliated to Soochow University, Suzhou, People’s Republic of China; f Department of Tuberculosis, Shandong Public Health Clinical Center, Jinan, People’s Republic of China; g Katharine Hsu International Research Center of Human Infectious Diseases, Shandong Public Health Clinical Center Affiliated to Shandong University, Jinan, People’s Republic of China; h Cepheid, Danaher Diagnostic Platform, Shanghai, People’s Republic of China

**Keywords:** Tuberculosis, MTB/RIF Ultra, accuracy, diagnosis, posterior oropharyngeal saliva

## Abstract

Background: Posterior oropharyngeal saliva (POS) is increasingly recognized as an alternative specimen for detecting respiratory pathogens. The accuracy of Xpert® MTB/RIF Ultra (X-Ultra), when performed on POS obtained from patients with paucibacillary pulmonary tuberculosis (TB) is unclear. Methods: We consecutively recruited adults with symptoms suggestive of pulmonary TB who were negative by both smear microscopy and Xpert MTB/RIF (X-Classic). Each participant was required to provide one bronchoalveolar lavage fluid (BALF) and one POS specimen, respectively. Diagnostic performances of X-Ultra and X-Classic on POS were compared against clinical and mycobacterial reference standards. Findings: 686 participants meeting inclusion criteria were consecutively enrolled into the study. The overall diagnostic sensitivities of X-Ultra and X-Classic on POS samples were 78.9% [95% confidence interval (CI): 72.8–83.8] and 56.4% (95% CI: 49.7–62.9), respectively; the specificities were 96.6% (95% CI: 94.3–98.1) for X-Ultra and 97.6 (95CI: 95.5–98.8) for X-Classic in POS specimens. Notably, the sensitivity of X-Ultra on POS was as sensitive as X-Classic on BALF against microbiological reference standard (78.9% VS 73.1%). Against clinical diagnosis as a reference standard, the sensitivities of X-Ultra and X-Classic on POS were 55.9% (95% CI: 50.5–61.2; 193/345) and 40.0% (95% CI: 34.8–45.4; 138/345), respectively. The risk of negative results with POS was dramatically increased with decreasing bacterial loads. Conclusions: The testing of POS using X-Ultra shows promise as a tool to identify patients with paucibacillary TB. Considering that bronchoscopy is a semi-invasive procedure, POS testing ahead of bronchoscopy, may decrease the need for bronchoscopic procedures, and the cost of care.

## Introduction

Tuberculosis (TB) caused by *Mycobacterium tuberculosis* (MTB) complex is the second cause of morbidity and mortality from a single infectious agent [1,2]. In 2020, an estimated 10.0 million persons had incident TB and 1.2 million TB-related deaths occurred worldwide [1] Early and accurate diagnosis to reduce risk of transmission is considered to be one of the most effective TB control strategies [3].

Sputum-based smear microscopy is commonly used to diagnose TB; however, it lacks sensitivity, especially for patients with paucibacillary TB [4,5]. Nucleic acid amplification technologies, such as the World Health Organization (WHO)-endorsed Xpert MTB/RIF (X-Classic) test (Cepheid, Sunnyvale, CA), represent a substantial advancement over smear microscopy in the diagnosis of TB [6,7]. Despite exhibiting great sensitivity, its performance remains suboptimal in paucibacillary specimens. In China, the proportion of active TB cases that present with paucibacillary sputum specimens is large and rising [8,9]. The sputum-negative or low bacterial load sputum patients typically undergo invasive sampling techniques, such as bronchoscopy, bronchoalveolar lavage or pulmonary biopsy, to increase the probability of detecting TB [4]. The use of invasive procedures is associated with nosocomial infection and increased mortality [10]. Hence, there is an important unmet clinical need for a noninvasive sample type with sufficient sensitivity to detect *M. tuberculosis* in respiratory specimens from these patients.

Posterior oropharyngeal saliva (POS) is increasingly recognized as an alternative respiratory specimen for detecting SARS-CoV-2 and other respiratory viruses [11,12]. It facilitates the diagnosis of COVID-19, given the simplicity of specimen collection, less exposure of healthcare workers and acceptable diagnostic performance. These findings raised interesting questions about whether POS could be used as a potential specimen for the diagnosis of pulmonary TB. However, no evidence has been presented to evaluate its utility in individuals with symptoms suggestive of pulmonary TB.

More recently, the Xpert MTB/RIF Ultra (X-Ultra) test (Cepheid, Sunnyvale, CA) was introduced to improve the sensitivity of TB diagnostics. The test incorporates a larger reaction tube compared to the MTB/RIF (X-Classic) and utilizes two multicopy amplification targets to detect MTB [13]. A prospective multicenter study among adult pulmonary TB patients demonstrated that X-Ultra outperformed X-Classic in the diagnosis of smear-negative TB patients [14]. The enhanced performance of the X-Ultra was also noted in extrapulmonary TB specimens [15]. This suggests that it may have potential clinical value in the diagnosis of paucibacillary TB using POS as a sample type. Here, we conducted a prospective multicenter study to determine the diagnostic accuracy of X-Ultra using POS for the diagnosis of paucibacillary pulmonary TB.

## Materials and methods

### Study design and participants

We conducted a prospective, multicenter, observational study to assess the performance of X-Ultra using POS samples for the diagnosis of pulmonary TB in participants with paucibacillary infection. Three hospitals participated in this study, including the Beijing Chest Hospital, Capital Medical University, the Fifth Hospital of Suzhou, and Shandong Public Health Clinical Center. Eligible study participants were adults who older than 18 years of age with radiographic abnormalities plus at least one symptom suggestive of pulmonary TB for enrollment, including a cough of 2 weeks or longer duration, hemoptysis, fever, chest pain, dyspnea, weight loss and/or night sweats. Three sputum specimens were collected from each participant for smear microscopy, mycobacterial culture, and X-Classic testing. After being screened for TB using smear microscopy and X-Classic, those participants with negative results by both methods who were willing to undergo bronchoalveolar lavage were included our prospective study. Baseline clinical characteristics were obtained at enrolment. Each participant was required to provide one bronchoalveolar lavage fluid and one POS specimen. Patients were excluded if bronchoalveolar lavage was contraindicated or informed consent was not given by the patient. The study was approved by the Ethics Committee of the Fifth Hospital of Suzhou (2020SKY018). This study was registered with the Chinese Clinical Trial Registry (ChiCTR, www.chictr.org.cn) under identifier ChiCTRINR-17012369.

### Sample size estimation

Sample size calculation was performed using the composite microbiological reference standard. The patients with a positive test (X-Classic, X-Ultra, or MGIT) for MTB from BALF were defined as definite TB. Assuming a sensitivity of 85% and specificity of 95%, we required 215 definite TB cases to yield 90% power with a set to 5%. Estimating the prevalence of definite TB would be 35%, we aimed to recruit 610 participants. To account for a potential 5% contamination rate for MGIT culture, 642 patients with symptoms suggestive of TB were included in our analysis.

### Procedures

At baseline, patients underwent smear microcopy, mycobacterial culture (mycobacteria growth indicator tube [MGIT] (manufacturer, City, Country)) and X-Classic testing of sputum. For patients with both negative smear and X-Classic results, a 6 mL of POS specimen and 10 ml of bronchoalveolar lavage fluid (BALF) was obtained from each patient. The POS samples were collected under the direct observation of clinicians in the early morning before mouth rinsing and breakfast before BAL samples done on the same day. The samples were tested by X-Ultra, X-Classic and MGIT following standard procedures as previously described [14]. POS samples were processed following the standardized protocols for BALF samples. The results of the smear microscopy, X-Classic (BALF and POS), X-Ultra and MGIT culture were reported to the attending clinicians, who decided on anti-TB treatment according to clinical guidelines. A follow-up visit 12 weeks after enrollment was conducted to assess the response to antituberculosis treatment based on symptoms and radiological findings. The patients received a final diagnosis of confirmed, clinically diagnosed or no pulmonary tuberculosis according to the published uniform case definition [16]. Patients with at least one positive MTB result via conventional culture method or molecular diagnostic method on BALF were defined as definite TB cases; whereas patients without any experimental diagnostic evidence, but who had exhibited a positive response to anti-TB therapy, were defined as clinically diagnosed TB cases. The bacterial load was quantified by X-Ultra assay on BALF specimen, including high, medium, low, extremely low, and trace. The *rpoB* Ct values were chosen to classify high, medium, low and extremely low levels of bacteria; while the trace category represented the paucibacillary samples which were IS*6110*/IS*1081* positive but *rpoB* negative.

### Statistical analysis

Diagnostic performances (sensitivity, specificity, and positive and negative predictive values) of X-Ultra, X-Classic, and MGIT culture were compared against a mycobacterial reference standard (definite TB by testing on BALF). Sensitivity was defined as the proportion of TB definite patients who tested positive by X-Ultra or X-Classic. Specificity was defined as the proportion of patients testing negative with any reference standard in BALF who tested negative by the X-Ultra or X-Classic. Additionally, diagnostic performances were compared against the clinical reference standards, in which the clinical outcomes of patients were evaluated at the week 12 visit. Test performances of X-Ultra and X-Classic for POS were compared with those of different diagnostics in BALF using the *χ*² test. Logistic regression analyses were conducted to identify factors associated with false-positive results by X-Ultra. The following variables were analysed: age, sex, residence, treatment history, comorbidity, clinical symptoms, and bacterial load in BALF. In addition, an exploratory analysis comparing X-Ultra for semi-quantification of bacterial loads into high, medium, low, very low and trace categories between POS and BALF was also done. Statistical analysis was done using the SPSS version 20.0 (IBM, Chicago, IL). A *p* value < 0.05 was considered as statistically significant.

### Role of the funding source

The funders of the study had no role in the study design, data collection, data analysis, data interpretation, or writing of the report. The corresponding author had full access to all the data in the study and had final responsibility for the decision to submit for publication.

## Results

### Study population

**Figure 1.**
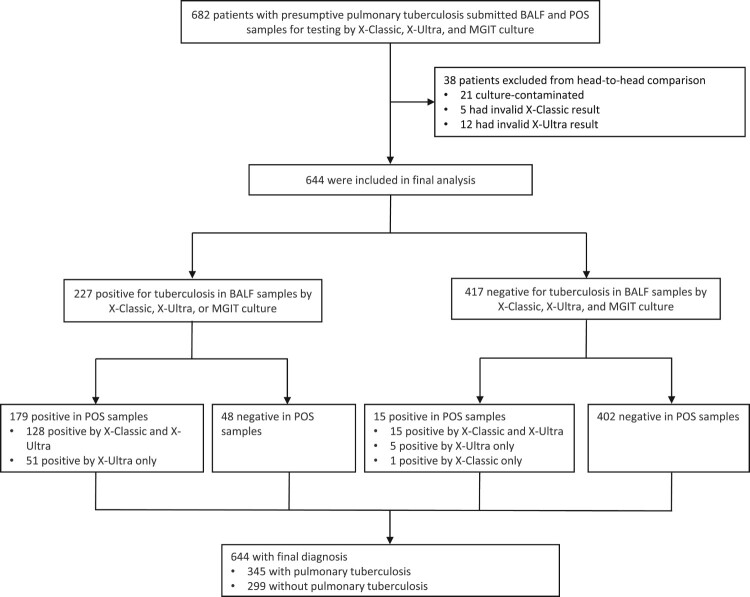
Trial profile. X-Ultra = Xpert MTB/RIF Ultra; X-Classic = Xpert MTB/RIF; MGIT = mycobacteria growth indicator tube; POS = posterior oropharyngeal saliva.

Between April 1, 2021 and March 31, 2022, 686 participants meeting inclusion criteria were consecutively enrolled into the study. A total of 38 participants were excluded from the analysis for a variety of reasons, including 21 who had culture-contaminated samples, 5 who had invalid X-Classic results, and 12 who had invalid X-Ultra results. Of 644 patients included in the analysis, 227 (35.2%) had positive evidence in BALF samples by X-Ultra or MGIT culture; and 417 (64.3%) had no laboratory evidence of TB, of whom 118 (28.3%) had clinically defined TB, and 299 (71.7%) had no clinical evidence of TB. Baseline demographic and clinical characteristics are shown in Table 1. The median age of patients at enrollment was 51.0 years [interquartile range (IQR): 33–63.75]. 57.1% of patients (370/644) were male. The most frequent comorbidities in this study were diabetes in 88 patients (13.7%) and COPD in 35 (5.4%). One patient (0.2%) had HIV and was included. Common primary clinical symptoms were cough (70.2%, 452/644) and fever (23.0%, 148/644) (Figure 1).

**Table 1.** Baseline characteristics of participants who underwent pulmonary tuberculosis testing.

	Pulmonary tuberculosis (N = 345)	Other pulmonary diseases (N = 299)	*p* value
**Age, year**	36.0 (28.0–55.0)	58.0 (48.0–68.0)	<0.001
≤44	214 (62.0)	59 (19.7)	
45–59	70 (20.3)	102 (34.1)	
≥60	61 (17.7)	138 (46.2)	
**Sex**			0.225
Female	156 (45.2)	121 (40.5)	
Male	189 (54.8)	178 (59.5)	
**Ethnicity**			0.515
Han	334 (96.8)	292 (97.7)	
Minority	11 (3.2)	7 (2.3)	
**Previous TB episode**	99 (28.7)	23 (7.7)	<0.001
**Smoke history**	76 (22.0)	100 (33.4)	0.001
**Comorbidities**			
COPD	3 (0.9)	32 (10.7)	<0.001
Bronchiectasia	8 (2.3)	22 (7.4)	0.002
Asthma	2 (0.6)	9 (3.0)	0.018
Diabetes	38 (11.0)	50 (16.7)	0.035
Hepatitis	12 (3.5)	10 (3.3)	0.926
HIV	1 (0.3)	0 (0.0)	>0.999
**Symptom**			
Cough	244 (70.7)	208 (69.6)	0.748
Hemoptysis	38 (11.0)	34 (11.4)	0.886
Fever	95 (27.5)	53 (17.7)	0.003
Weight loss	38 (11.0)	37 (12.4)	0.592

### Sensitivity and specificity

Among patients with definite TB, the overall diagnostic sensitivities of X-Ultra and X-Classic on POS samples were 78.9% [95% confidence interval (CI): 72.8–83.8] and 56.4% (95% CI: 49.7–62.9), respectively. The X-Ultra assay was significantly more sensitive than X-Classic (*p *< 0.001). Against a clinical diagnosis reference standard, the sensitivities were 55.9% (95% CI: 50.5–61.2) for X-Ultra and 40.0 (95% CI: 34.8–45.4) for X-Classic in POS specimens; whereas the sensitivities were 64.1% (95% CI: 58.7–69.1) for X-Ultra, 48.1% (95% CI: 42.8–53.5) for X-Classic in BALF specimens. Statistical analysis revealed that X-Ultra was more sensitive than X-Classic on both POS and BALF samples (*p *< 0.001). Notably, the sensitivity of X-Ultra on POS was as sensitive as X-Classic on BALF against microbiological reference standard (78.9% vs. 73.1%) (Table 2).

**Figure 2.**
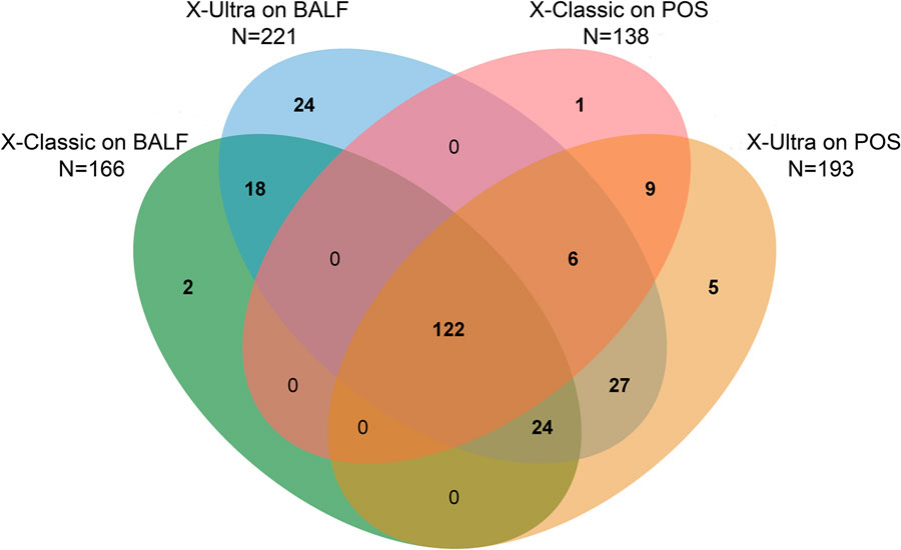
Venn diagram of overlap in paucibacillary tuberculosis on POS and BALF. X-Ultra = Xpert MTB/RIF Ultra; X-Classic = Xpert MTB/RIF; MGIT = mycobacteria growth indicator tube; POS = posterior oropharyngeal saliva; BALF = bronchoalveolar lavage fluid; AUC = Area Under Curve.

We further analysed the distribution and overlap of positive POS and BALF by X-Ultra and X-Classic. In total, 238 (37.0%) were bacteriologically confirmed on BALF and POS samples together, of which 24 (10.1%) were exclusively detected with X-Ultra on BALF. Of the 227 (95.4%) patients with positive BALF, 138 (60.1%) and 193 (85.0%) were also diagnosed with X-Classic and X-Ultra on POS samples, respectively. In addition, there were 5 (2.1%) and 1 (0.4%) positive result exclusively by X-Ultra and X-Classic on POS, respectively (Figure 2).

**Figure 3.**
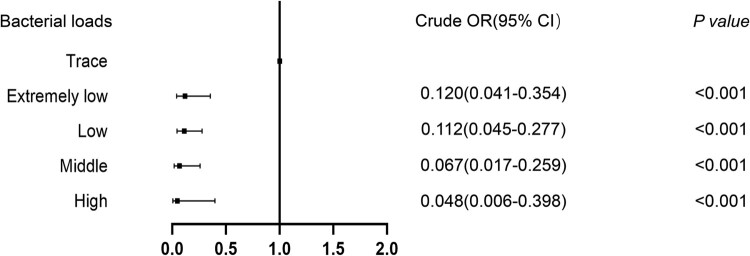
Relative risk of false-negative X-Ultra results on POS samples stratified by bacterial load. X-Ultra = Xpert MTB/RIF Ultra; POS = posterior oropharyngeal saliva; OR = odds ratio. The bacterial load is quantified by X-Ultra assay on BALF specimen.

Univariable of factors predicting bacteriological confirmation of pulmonary TB on POS are summarized in Table S1. The risk of negative bacteriological results was dramatically increased with decreasing bacterial loads (Figure 3). By contrast, all other factors were non-predictive in the analysis.

### Quantification of bacterial loads

Of the 193 (39.0%) positive results by X-Ultra on POS, 10 (5.2%), 29 (15.0%), 63 (32.6%), 46 (23.8%) and 45 (23.3%) had high, medium, low, very low, and trace call results. The numbers of samples with high, medium, low, and very low numbers of bacteria were 17 (7.7%), 37 (16.7%), 86 (38.9%), 44 (19.9%) and 37 (16.7%) by X-Classic. Of 56 POS samples positive by X-Ultra but negative by X-Classic, 43 (76.8%) were categorized as containing trace numbers of bacteria, suggesting that the samples giving trace results significantly contributed to the increased sensitivity of X-Ultra in comparison to X-Classic (Table 2).

**Table 2.** Diagnostic performance of X-Ultra and X-Classic against reference standard.

Methods	BALF X-Classic	BALF X-Ultra	POS X-Classic	POS X-Ultra
**Reference standard: definite pulmonary TB**				
Positive test	166/227	221/227	128/227	179/227
Sensitivity	73.1(66.8–78.7)	97.4(94.1–98.9)	56.4(49.7–62.9)	78.9(72.8–83.8)
Specificity	100.0(98.9–100.0)	100.0(98.9–100.0)	97.6(95.5–98.8)	96.6(94.3–98.1)
PPV	100.0(97.2–100.0)	100.0(97.9–100.0)	92.8(86.7–96.3)	92.7(87.9–95.8)
NPV	87.2(83.8–90.0)	98.6(96.8–99.4)	80.4(76.6–83.7)	89.4(86.0–92.0)
AUC	86.6(83.0–90.1)	98.7(97.5–99.9)	77.0(72.7–81.3)	87.7(84.4–91.1)
**Reference standard: definite and clinically diagnosed pulmonary TB**				
Positive test	166/345	221/345	138/345	193/345
Sensitivity	48.1 (42.8–53.5)	64.1 (58.7–69.1)	40.0 (34.8–45.4)	55.9 (50.5–61.2)
Specificity	100.0 (98.4–100.0)	100.0 (98.4–100)	100.0 (98.4–100.0)	100.0 (98.4–100.0)
PPV	100.0 (97.2–100.0)	100.0 (97.9–100.0)	100.0 (96.6–100.0)	100.0 (97.6–100.0)
NPV	62.6 (58.0–66.9)	70.7 (66.1–75.0)	59.1 (54.7–63.4)	66.3 (61.7–70.6)
AUC	74.1 (70.2–77.9)	82.0 (78.7–85.4)	70.0 (66.0–74.0)	78.0 (74.4–81.6)

Notes: Data in parentheses are 95% CIs. X-Ultra = Xpert MTB/RIF Ultra; X-Classic = Xpert MTB/RIF; PPV = positive predictive value; NPV = negative predictive value.

## Discussion

This study prospectively assessed the diagnostic accuracy of the Xpert MTB-RIF Ultra test for the rapid detection of paucibacillary pulmonary tuberculosis using POS samples. Our data showed that testing POS samples with the X-Ultra was a useful alternative to BALF in patients with low bacterial loads in sputum. In China and other resource-limited settings, the application of rapid diagnostic tests significantly improves the detection of active pulmonary TB cases [17]; however, a substantial proportion of TB patients remain undiagnosed due to the absence of laboratory evidence [1]. In resource-rich settings, bronchoalveolar lavage fluid and fine needle aspirate of the lung are routinely performed for patients with symptoms suggestive of TB who have negative results by sputum-based assays to increase diagnostic yield [4,18]. Unfortunately, the infrastructure and trained personnel required for bronchoscopy make it inaccessible for patients in resource-limited settings. In addition, bronchoscopy is not suitable for all patients due to its invasive nature [19]. The implementation of molecular testing using POS samples could provide the means to identify the presence of tubercle bacilli in a safe and comfortable manner. The automated X-Ultra test enables the use of advanced molecular testing procedures in primary healthcare centres. By replacing bronchoscopy with POS testing, or at least, using POS ahead of bronchoscopy, the risk of invasive bronchoscopy and the associated cost could be decreased.

Many studies have compared the results of induced sputum tests with bronchoscopy in individuals with presumed active pulmonary TB patients [10,20]. These studies showed that the testing of induced sputum samples yielded comparable to superior performance when compared to the testing of samples obtained bybronchoscopy [10]. Despite being a potential alternative to BALF, the routine use of sputum induction is hampered by the significant resource requirements required for specific training, equipment, and enhanced infection control practices. The collection of POS samples offsets theses disadvantages, and reduces the need for a complicated testing infrastructure, by providing a more practical diagnostic specimen type for the detection of tuberculosis.

In a previous report by Shenai and coresearchers, the X-Classic showed a lower sensitivity in detecting MTB from saliva specimens compared with testing on sputum samples isolated from the same patients [21]. However, a recent study conducted in Uganda revealed that X-Ultra on a single saliva sample produced a sensitivity of 90% relative to the composite MGIT reference standard [22]. The use of X-Ultra and enrollment of only individuals with definite TB may explain the promising sensitivity of X-Ultra on saliva specimens in the latter study. Given the paucibacillary population, X-Ultra on POS samples led to an even better sensitivity in the present study, emphasizing that POS appears to be a feasible specimen for TB diagnosis.

Previous studies have suggested that X-Ultra is significantly more sensitive than X-Classic for the diagnosis of pulmonary tuberculosis in both HIV-infected and HIV-naïve individuals [14,23]. Similarly, we also found that X-Ultra on POS samples produced higher sensitivity than X-Classic, indicating that X-Ultra initial test for presumed pulmonary tuberculosis patients using POS samples, especially patients without any positive laboratory TB evidence. Although the appearance of cough is more likely to be associated with higher bacteria load in clinical specimens, many asymptomatic patients, recognized as subclinical TB patients, also expel aerosolized tubercle bacilli and may infect susceptible individuals in the community [24,25]. In a recent meta-analysis, the median percentage of subclinical TB cases was 50.4% across included surveys [25]. A retrospective study also revealed that about one-third of prevalent clinically confirmed TB was subclinical in China [9]. A significant proportion of subclinical TB within the global TB burden limits the impact of current diagnostic strategies for TB and has prompted the call for the development of novel tests capable of identifying subclinical TB. However, new diagnostic development and validation are time intensive which has led to the exploration of other sample types to improve disease detection. Symptom-agnostic screening through chest X-ray and X-Ultra on POS provides an opportunity to improve the detection of subclinical TB, thereby benefitting individuals by preventing disease progression and the community by reducing transmission.

Notably, both X-Ultra and X-Classic had a specificity of 100% on POS samples when clinical reference standards were used. These results are in line with findings by Donovan and colleagues that showed that X-Ultra had promising specificity in the diagnosis of tuberculous meningitis [26]. By contrast, a previous study by Dorman et al. demonstrated that the increased sensitivity of X-Ultra resulted in a loss of specificity, especially for patients with a medical history of anti-TB treatment [14]. The X-Ultra-positive, culture-negative results were majorly attributed to individuals with previous TB episode, which reduced confidence in trace-positive results by X-Ultra. Considering that the exclusion of X-Ultra trace results would result in the loss of sensitivity benefits of X-Ultra in comparison to X-Classic [27], our primary data suggest that inclusion is a more preferable strategy for dealing with trace results from POS specimens.

In our study, approximate one-fifth of definite TB cases had false-negative X-Ultra results on POS samples. A major explanation for these false-negative results is due to the extremely low mycobacterial burden in the respiratory tract specimens, as illustrated by our analysis that demonstrated that the initial bacterial load was the sole predictor of X-Ultra false negativity. Another concern regarding low bacterial load in POS samples was that approximate one quarter of samples yielded indeterminate results for RIF susceptibility due to their paucibacillary nature. In addition, increasing evidence suggests that MTB isolates with a very low bacterial load were most frequently misdiagnosed as RIF-resistant by X-Classic [28,29]. These findings highlight a sustained need for more sensitive tests of the presence of MTB and its RIF susceptibility for paucibacillary pulmonary TB patients.

There are several limitations to the present study. First, diagnostic accuracy estimates using POS samples were obtained through the testing of HIV-negative adults with the exception of one participant. Additional data are required to examine the performance of X-Ultra using POS from adults and children living with HIV given the paucibacillary nature of TB disease in these populations. Second, the copy number of the multicopy amplification target *IS*6110 exhibits great diversity across different MTB lineages, which may affect the diagnostic sensitivity [30]. Although our study was done in three tertiary referral centres, all pilots are located in China where L2 lineage is predominant [31]. Further evaluation is urgently needed to validate our results, particularly in regions where other lineages are epidemic. Third, due to paucibacillary nature of study population, a substantial number of TB patients yielded negative cultures, thus limiting further detection of *in vitro* RIF susceptibility. As a consequence, it is difficult to clarify if false-positive RIF mutation results existed in POS samples. Finally, POS samples were only employed in X-Ultra and X-Classic analysis rather than MGIT culture, which hampers us to assess the feasibility of POS for mycobacterial culture at these facilities.

In summary, we are the first to report the use of the Xpert MTB-RIF Ultra test on posterior oropharyngeal saliva specimens and show its excellent diagnostic accuracy for patients with paucibacillary TB. Considering that bronchoscopy is a semi-invasive procedure, replacing it with POS testing, at least using POS ahead of bronchoscopy, decreases the risk from bronchoscopic procedures and associated cost.

## Supplementary Material

Supplemental Material

## References

[1] WHO. Global tuberculosis report 2021. Geneva: World Health Organization; 2021.

[2] Floyd K, Glaziou P, Zumla A, et al. The global tuberculosis epidemic and progress in care, prevention, and research: an overview in year 3 of the end TB era. Lancet Respir Med. 2018;6(4):299–314. 29595511 10.1016/S2213-2600(18)30057-2

[3] Lin HH, Dowdy D, Dye C, et al. The impact of new tuberculosis diagnostics on transmission: why context matters. Bull World Health Organ. 2012;90(10):739–747A. 23109741 10.2471/BLT.11.101436 PMC3471051

[4] Theron G, Peter J, Meldau R, et al. Accuracy and impact of Xpert MTB/RIF for the diagnosis of smear-negative or sputum-scarce tuberculosis using bronchoalveolar lavage fluid. Thorax. 2013;68(11):1043–1051. 23811536 10.1136/thoraxjnl-2013-203485 PMC5523966

[5] Steingart KR, Ng V, Henry M, et al. Sputum processing methods to improve the sensitivity of smear microscopy for tuberculosis: a systematic review. Lancet Infect Dis. 2006;6(10):664–674. 17008175 10.1016/S1473-3099(06)70602-8

[6] MacLean E, Kohli M, Weber SF, et al. Advances in molecular diagnosis of tuberculosis. J Clin Microbiol. 2020;58(10):e01582-19. 32759357 10.1128/JCM.01582-19 PMC7512154

[7] WHO. Xpert MTB/RIF assay for the diagnosis of pulmonary and extrapulmonary TB in adults and children: policy update. Geneva: World Health Organization; 2013. 25473701

[8] Wang L, Zhang H, Ruan Y, et al. Tuberculosis prevalence in China, 1990-2010; a longitudinal analysis of national survey data. Lancet. 2014;383(9934):2057–2064. 24650955 10.1016/S0140-6736(13)62639-2

[9] Tang P, Liang E, Zhang X, et al. Prevalence and risk factors of subclinical tuberculosis in a low-incidence setting in China. Front Microbiol. 2021;12:731532. 35087480 10.3389/fmicb.2021.731532 PMC8787132

[10] McWilliams T, Wells AU, Harrison AC, et al. Induced sputum and bronchoscopy in the diagnosis of pulmonary tuberculosis. Thorax. 2002;57(12):1010–1014. 12454293 10.1136/thorax.57.12.1010 PMC1758793

[11] To KK, Tsang OT, Leung WS, et al. Temporal profiles of viral load in posterior oropharyngeal saliva samples and serum antibody responses during infection by SARS-CoV-2: an observational cohort study. Lancet Infect Dis. 2020;20(5):565–574. 32213337 10.1016/S1473-3099(20)30196-1 PMC7158907

[12] Wong SCY, Tse H, Siu HK, et al. Posterior oropharyngeal saliva for the detection of severe acute respiratory syndrome coronavirus 2 (SARS-CoV-2). Clin Infect Dis. 2020;71(11):2939–2946. 32562544 10.1093/cid/ciaa797 PMC7337706

[13] Chakravorty S, Simmons AM, Rowneki M, et al. The New Xpert MTB/RIF Ultra: Improving Detection of Mycobacterium tuberculosis and Resistance to Rifampin in an Assay Suitable for Point-of-Care Testing. mBio. 2017;8(4):e00812-17. 28851844 10.1128/mBio.00812-17 PMC5574709

[14] Dorman SE, Schumacher SG, Alland D, et al. Xpert MTB/RIF Ultra for detection of mycobacterium tuberculosis and rifampicin resistance: a prospective multicentre diagnostic accuracy study. Lancet Infect Dis. 2018;18(1):76–84. 29198911 10.1016/S1473-3099(17)30691-6 PMC6168783

[15] Kohli M, Schiller I, Dendukuri N, et al. Xpert MTB/RIF Ultra and Xpert MTB/RIF assays for extrapulmonary tuberculosis and rifampicin resistance in adults. Cochrane Database Syst Rev. 2021;1:CD012768. 33448348 10.1002/14651858.CD012768.pub3 PMC8078545

[16] Sossen B, Broger T, Kerkhoff AD, et al. “SILVAMP TB LAM” rapid urine tuberculosis test predicts mortality in patients hospitalized with human immunodeficiency virus in South Africa. Clin Infect Dis. 2020;71(8):1973–1976. 31917832 10.1093/cid/ciaa024 PMC8240995

[17] Jacobson KR, Sabin LL. Scaling up multidrug-resistant tuberculosis care in China. Lancet Glob Health. 2015;3(4):e183–e184. 25794667 10.1016/S2214-109X(15)70093-8 PMC4492310

[18] Achkar JM, Lawn SD, Moosa MY, et al. Adjunctive tests for diagnosis of tuberculosis: serology, ELISPOT for site-specific lymphocytes, urinary lipoarabinomannan, string test, and fine needle aspiration. J Infect Dis. 2011;204(Suppl 4):S1130–S1141. 21996695 10.1093/infdis/jir450 PMC3192548

[19] Brown M, Varia H, Bassett P, et al. Prospective study of sputum induction, gastric washing, and bronchoalveolar lavage for the diagnosis of pulmonary tuberculosis in patients who are unable to expectorate. Clin Infect Dis. 2007;44(11):1415–1420. 17479935 10.1086/516782

[20] Hepple P, Ford N, McNerney R. Microscopy compared to culture for the diagnosis of tuberculosis in induced sputum samples: a systematic review. Int J Tuberc Lung Dis. 2012;16(5):579–588. 22410498 10.5588/ijtld.11.0617

[21] Shenai S, Amisano D, Ronacher K, et al. Exploring alternative biomaterials for diagnosis of pulmonary tuberculosis in HIV-negative patients by use of the GeneXpert MTB/RIF assay. J Clin Microbiol. 2013;51(12):4161–4166. 24108610 10.1128/JCM.01743-13 PMC3838083

[22] Byanyima P, Kaswabuli S, Musisi E, et al. Feasibility and sensitivity of saliva GeneXpert MTB/RIF Ultra for tuberculosis diagnosis in adults in Uganda. Microbiol Spectr. 2022;10(5):e0086022. 36154664 10.1128/spectrum.00860-22 PMC9603304

[23] Mishra H, Reeve BWP, Palmer Z, et al. Xpert MTB/RIF Ultra and Xpert MTB/RIF for diagnosis of tuberculosis in an HIV-endemic setting with a high burden of previous tuberculosis: a two-cohort diagnostic accuracy study. Lancet Respir Med. 2020;8(4):368–382. 32066534 10.1016/S2213-2600(19)30370-4

[24] Kendall EA, Shrestha S, Dowdy DW. The epidemiological importance of subclinical tuberculosis. A critical reappraisal. Am J Respir Crit Care Med. 2021;203(2):168–174. 33197210 10.1164/rccm.202006-2394PP PMC7874405

[25] Frascella B, Richards AS, Sossen B, et al. Subclinical tuberculosis disease-a review and analysis of prevalence surveys to inform definitions, burden, associations, and screening methodology. Clin Infect Dis. 2021;73(3):e830–ee41. 32936877 10.1093/cid/ciaa1402 PMC8326537

[26] Donovan J, Thu DDA, Phu NH, et al. Xpert MTB/RIF Ultra versus Xpert MTB/RIF for the diagnosis of tuberculous meningitis: a prospective, randomised, diagnostic accuracy study. Lancet Infect Dis. 2020;20(3):299–307. 31924551 10.1016/S1473-3099(19)30649-8 PMC7045088

[27] Opota O, Zakham F, Mazza-Stalder J, et al. Added value of Xpert MTB/RIF Ultra for diagnosis of pulmonary tuberculosis in a low-prevalence setting. J Clin Microbiol. 2019;57(2):e01717-18. 30541937 10.1128/JCM.01717-18 PMC6355522

[28] Huo F, Ma Y, Liu R, et al. Interpretation of discordant rifampicin susceptibility test results obtained using GeneXpert vs phenotypic drug susceptibility testing. Open Forum Infect Dis. 2020;7(8):ofaa279. 32766385 10.1093/ofid/ofaa279 PMC7397830

[29] Ocheretina O, Byrt E, Mabou MM, et al. False-positive rifampin resistant results with Xpert MTB/RIF version 4 assay in clinical samples with a low bacterial load. Diagn Microbiol Infect Dis. 2016;85(1):53–55. 26915638 10.1016/j.diagmicrobio.2016.01.009 PMC4841693

[30] Roychowdhury T, Mandal S, Bhattacharya A. Analysis of IS6110 insertion sites provide a glimpse into genome evolution of mycobacterium tuberculosis. Sci Rep. 2015;5:12567. 26215170 10.1038/srep12567 PMC4517164

[31] Pang Y, Zhou Y, Zhao B, et al. Spoligotyping and drug resistance analysis of mycobacterium tuberculosis strains from national survey in China. PLoS One. 2012;7(3):e32976. 22412962 10.1371/journal.pone.0032976 PMC3296750

