# Supplementary material for: Accuracy of Xpert® MTB/RIF Ultra test for posterior oropharyngeal saliva for the diagnosis of paucibacillary pulmonary tuberculosis: a prospective multicenter study
Source: Emerg Microbes Infect. 2022 Dec 12;12(1):2148564. doi: 10.1080/22221751.2022.2148564 (PMC9754037; doi:10.1080/22221751.2022.2148564)
Supplement: Supplemental Material [file TEMI_A_2148564_SM6096.docx]

| **Table S1 Factors associated with tests among the patients enrolled in this study** | | | | | |  |  |
| --- | --- | --- | --- | --- | --- | --- | --- |
| **Characteristics** | **True positive** | | **False negative** | | **Total** | | **Crude OR (95% CI)** |
|  | **No.** | **Col %** | **No.** | **Col %** | **No.** | **Col %** |  |
| **Age (year)** |  |  |  |  |  |  |  |
| ≤44 | 105 | 58.7 | 29 | 60.4 | 134 | 59.0 |  |
| 45-59 | 37 | 20.7 | 11 | 22.9 | 48 | 21.1 | 1.076(0.489-2.369) |
| ≥60 | 37 | 20.7 | 8 | 16.7 | 45 | 19.8 | 0.783(0.329-1.864) |
| **Gender** |  |  |  |  |  |  |  |
| Female | 81 | 45.3 | 25 | 52.1 | 106 | 46.7% |  |
| Male | 98 | 54.7 | 23 | 47.9 | 121 | 53.3% | 0.76(0.402-1.440) |
| **Smoke** |  |  |  |  |  |  |  |
| No | 133 | 74.3 | 40 | 83.3 | 173 | 76.2% |  |
| Yes | 46 | 25.7 | 8 | 16.7 | 54 | 23.8% | 0.578(0.252-1.326) |
| **Ethnic** |  |  |  |  |  |  |  |
| Han nationality | 172 | 96.1 | 45 | 93.8 | 217 | 95.6% |  |
| Minority nationality | 7 | 3.9 | 3 | 6.3 | 10 | 4.4% | 1.638(0.407-6.588) |
| **History of Pulmonary TB** |  |  |  |  |  |  |  |
| No | 124 | 69.3 | 27 | 56.3 | 151 | 66.5% |  |
| Yes | 55 | 30.7 | 21 | 43.8 | 76 | 33.5% | 1.754(0.913-3.368) |
| **Diabetes** |  |  |  |  |  |  |  |
| No | 152 | 84.9 | 42 | 87.5 | 194 | 85.5% |  |
| Yes | 27 | 15.1 | 6 | 12.5 | 33 | 14.5% | 0.804(0.312-2.076) |
| **Hepatitis** |  |  |  |  |  |  |  |
| No | 174 | 97.2 | 43 | 89.6 | 217 | 95.6% |  |
| Yes | 5 | 2.8 | 5 | 10.4 | 10 | 4.4% | 4.047(1.121-14.608) |
| **Cough** |  |  |  |  |  |  |  |
| No | 30 | 16.8 | 14 | 29.2 | 44 | 19.4% |  |
| Yes | 149 | 83.2 | 34 | 70.8 | 183 | 80.6% | 0.489(0.234-1.02) |
| **Hemoptysis** |  |  |  |  |  |  |  |
| No | 162 | 90.5 | 40 | 83.3 | 202 | 89.0% |  |
| Yes | 17 | 9.5 | 8 | 16.7 | 25 | 11.0% | 1.906(0.768-4.729) |
| **Fever** |  |  |  |  |  |  |  |
| No | 119 | 66.5 | 32 | 66.7 | 151 | 66.5% |  |
| Yes | 60 | 33.5 | 16 | 33.3 | 76 | 33.5% | 0.992(0.505-1.949) |
| **Weight loss** |  |  |  |  |  |  |  |
| No | 153 | 85.5 | 43 | 89.6 | 196 | 86.3% |  |
| Yes | 26 | 14.5 | 5 | 10.4 | 31 | 13.7% | 0.684(0.248-1.888) |
